# Association Between Contact Force and Acute Lesion Effectiveness in Focal Pulsed Field Ablation

**DOI:** 10.1111/jce.70391

**Published:** 2026-06-01

**Authors:** Sara Poggi, Nandor Szegedi, Cheryl Teres, Daniel Scherr, Christian Heeger, Gabor Szeplaki, Benjamin Berte, Alexandra Karsai, Teresa Strisciuglio, Giuseppe Stabile

**Affiliations:** ^1^ Mediterranea Cardiocentro Napoli Italy; ^2^ IRCCS I.N.M. Neuromed Pozzilli Italy; ^3^ Semmelweis University Budapest Hungary; ^4^ Lausanne University Hospital (CHUV) Lausanne Switzerland; ^5^ Medical University Graz Austria; ^6^ Asklepios Clinic Altona Hamburg Germany; ^7^ Mater Misericordie University Hospital Dublin Ireland; ^8^ Hirslanden Klinik St Anna Lucerne Switzerland; ^9^ Università Federico II Napoli Italy

**Keywords:** atrial fibrillation, catheter ablation, contact force, impedance drop, pulsed filed energy

## Abstract

**Introduction:**

Pulsed field ablation (PFA), based on irreversible electroporation, is a non‐thermal modality designed to create myocardial lesions while minimizing collateral injury. The determinants of acute lesion effectiveness in humans remain incompletely understood. We evaluated the association of contact force (CF) and impedance drop (ID) with acute lesion effectiveness during focal PFA index‐guided pulmonary vein isolation (PVI).

**Methods and Results:**

Twenty‐four patients undergoing PFA‐guided PVI for atrial fibrillation (AF) ablation were included. Electroanatomical mapping was performed in sinus rhythm using high‐density catheters. Ablation was delivered using a CF catheter in a point‐by‐point workflow guided by a PFA index. Lesion effectiveness was defined by acute electrophysiological criteria. CF, ID, and PFA index achievement were analyzed at each ablation point. A total of 1460 ablation points were analyzed (1407 successful, 53 unsuccessful). Median CF was significantly higher in successful compared with unsuccessful lesions (15 vs. 11.3 g, *p* < 0.001). A CF value of 8 g yielded high sensitivity (90.3%) but low specificity (38.9%) for lesion effectiveness. ID did not differ between groups (*p* = 0.58). Target PFA index achievement was more frequent in successful lesions (67% vs. 9.4%, *p* < 0.01).

**Conclusions:**

CF is a significant but imperfect predictor of acute lesion effectiveness in PFA, since averaged CF values may inadequately reflect the instantaneous catheter–tissue interaction at the moment of pulse delivery. These findings support a paradigm in which maintaining sufficient real‐time CF, rather than achieving a fixed average threshold, is critical for effective lesion formation.

## 1 Introduction

Pulsed field ablation (PFA) is a largely non‐thermal ablation modality, based on irreversible electroporation, designed to create effective myocardial lesions while minimizing the risk of collateral tissue injury [1]. Although several biophysical parameters are thought to influence lesion formation, their relative contribution to acute lesion effectiveness in humans has not been clearly elucidated. The present study aimed to evaluate the association of contact force (CF) and impedance drop (ID) with acute lesion effectiveness during focal PFA index‐guided pulmonary vein isolation (PVI).

## 2 Methods

This was a prospective multicenter study including patients undergoing first‐time PVI for atrial fibrillation (AF) ablation. Electroanatomical mapping was performed in sinus rhythm with a high‐density catheter (PentaRay Nav or OctaRay Nav, Johnson & Johnson MedTech Inc, Irwindale, CA, USA). PFA ablation was performed using a quadripolar catheter (ThermoCool SmartTouch SF‐Dual Energy, Johnson & Johnson MedTech Inc, Irwindale, CA), featuring a 3.5 mm saline irrigated tip electrode with an integrated magnetic CF sensor and an internal thermocouple. Ablation was performed using a PFA index‐guided point‐by‐point workflow using real‐time automated lesion tagging, with a maximum inter‐lesion distance of ≤ 6 mm, with target index values of 400 for the posterior and inferior wall and 500−550 for the anterior and superior wall [2]. PF applications were delivered sequentially to create circumferential lesions around the proximal PV ostia or ipsilateral veins according to the left atrial anatomy or the operator's preference. In general, overlapping lesions were not performed. Upon completion of circumferential ablation, PVI was assessed with the high‐density mapping catheter by verification of entrance and exit block (first‐pass isolation). In cases of non‐first pass, touch‐up ablation was delivered with PFA or radiofrequency (RF) energy according to operator discretion.

For the purpose of this analysis, lesion effectiveness was assessed using acute functional electrophysiological criteria. Unsuccessful lesions were defined as ablation sites exhibiting persistent local electrogram voltage and continued ability to capture tissue following energy delivery. For each ablation point, procedural parameters including CF, ID, and achievement of the target PFA index were recorded and analyzed.

## 3 Results

**Figure 1:**
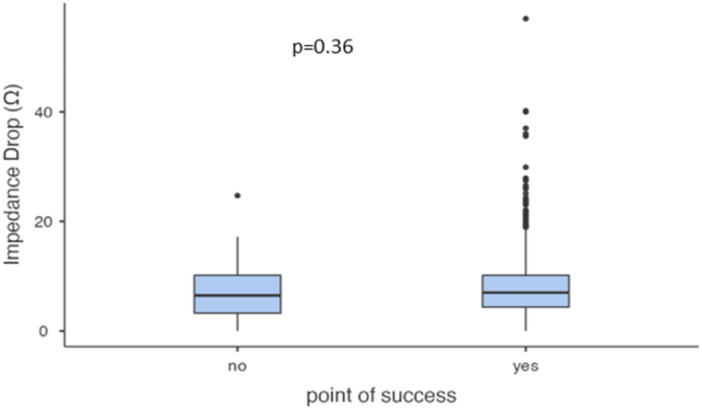
Median impedance drop at failure and successful ablation points.

**Figure 2:**
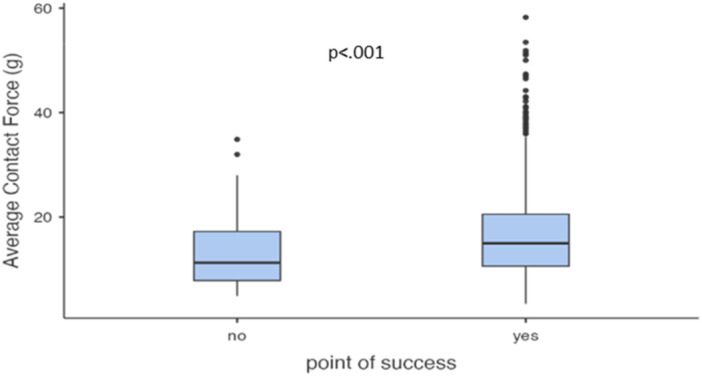
Median average contact force at failure and successful ablation points.

**Figure 3:**
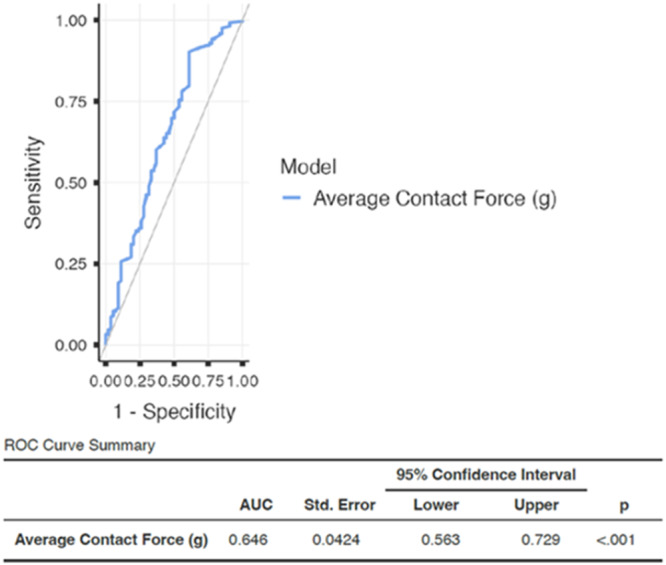
Receiver operating characteristic curve of average contact force.

Twenty‐four patients were included (17 with paroxysmal AF, 7 with persistent AF), with a first‐pass PV isolation rate of 84%. Sixteen PV gaps were identified. At the end of the procedures, all PVs had been successfully isolated in all study patients. A total of 1460 PFA points were analyzed at the lesion level, including 1407 successful and 53 unsuccessful ablation points. The target PFA index was reached in 5 of 53 (9.4%) unsuccessful ablation points and in 945 of 1407 (67%) of successful ablation points (*p *< 0.01). Median ID did not differ between successful and unsuccessful ablation points (7 [4.4−10.2] vs. 6.5 Ω [IQR 3.3−10.2], respectively, *p* = 0.58) (Figure 1). In contrast, median CF was higher at successful ablation points (15 [10.6−20.6] vs. 11.3 g [7.9−17.3], *p* < 0.001) (Figure 2). Receiver operating characteristic curve analysis yielded an area under the curve of 0.65 for a CF value of 8 g, corresponding to a sensitivity 90.3%, a specificity 38.9%, and an accuracy of 88.4% (Figure 3).

## 4 Discussion

This study evaluated the association between selected biophysical parameters and acute functional lesion effectiveness during focal PFA index‐guided PVI. The main findings were that: (1) Higher CF is associated with lesion effectiveness; (2) ID did not discriminate between successful and unsuccessful ablation points; (3) achievement of the target PFA index was associated with successful PFA ablation; (4) averaged CF measurements may inadequately reflect effective tissue contact in PFA.

In RF‐based ablation, lesion size and transmurality are influenced by catheter stability, CF, power output, temperature, and application duration [3, 4, 5]. In contrast to RF ablation, which relies on time‐dependent thermal injury, PFA delivers ultra‐short pulses over milliseconds. This fundamental mechanistic difference suggests that the factors governing lesion formation may differ substantially between the two modalities. In a swine model, Nakagawa et al. [2] demonstrated that a PFA index incorporating CF and the number of PFA pulses correlates with lesion depth in real‐time, highlighting the importance of adequate electrode‐tissue contact for irreversible cardiomyocyte electroporation. In contrast, intracardiac electrogram attenuation, ID, and electrode temperature were poor predictors of PFA lesion size in experimental settings. Human data addressing the biophysical determinants of PFA lesion effectiveness remain limited. In the present study, higher CF was associated with acute functional lesion effectiveness. Although a CF value around 8 g was associated with high sensitivity, the modest discriminatory performance and limited specificity indicate a continuous, probabilistic relationship rather than a discrete CF threshold. Accordingly, CF should be viewed as one contributor to acute lesion effectiveness rather than a deterministic criterion. A key consideration when interpreting CF in PFA is the temporal mismatch between force measurement and energy delivery. Unlike RF ablation, where energy is delivered continuously over several seconds, PFA consists of ultra‐short pulses delivered over milliseconds. As a result, the instantaneous CF at the moment of pulse delivery, rather than the time‐averaged CF, may be the critical determinant of lesion formation. Cardiac motion and respiration introduce significant beat‐to‐beat variability in catheter–tissue contact, such that an average CF of 8 g may include periods of minimal or absent contact during which PFA pulses are delivered. Consequently, averaged CF values may overestimate effective tissue contact at the critical moment of energy delivery. Higher average CF values may increase the likelihood that sufficient instantaneous contact is maintained during pulse delivery. These findings support a ‘minimum CF’ hypothesis in PFA, whereby lesion formation depends on maintaining a critical level of electrode–tissue contact at the precise moment of pulse delivery. Achieving a higher average CF may therefore be necessary not to increase lesion size per se, but to mitigate transient losses of contact caused by cardiac motion. This concept may explain why a CF value of 8 g demonstrated high sensitivity but limited specificity, reflecting a continuous relationship rather than a discrete threshold. This paradigm differs fundamentally from RF ablation, where continuous energy delivery over several seconds allows for temporal averaging of contact conditions, rendering lesion formation more tolerant to transient reductions in CF. Future studies incorporating high‐resolution CF data synchronized with PFA delivery are needed to better characterize the relationship between instantaneous CF and lesion formation.

During RF catheter ablation, an ID ≥ 10 Ω has traditionally been considered as a marker of adequate lesion formation [6, 7, 8]. In the present study, ID did not discriminate between successful and unsuccessful ablation points. This is consistent with experimental observations indicating that impedance changes during PFA primarily reflect membrane permeability alterations rather than irreversible tissue injury [9]. Thus, ID appears to have limited mechanistic and clinical relevance in the assessment of PFA lesion effectiveness.

Our analysis is restricted to acute functional endpoints and does not capture potentially reversible or late‐recovering lesions, which may have different biophysical determinants. Future studies incorporating systematic remapping would provide a more comprehensive assessment of lesion durability.

## 5 Conclusion

In conclusion, CF is a significant but imperfect predictor of acute lesion effectiveness. Conventional time‐averaged CF measurements may not adequately capture the critical catheter–tissue interaction at the moment of energy delivery. A paradigm shift toward understanding real‐time contact dynamics may be required to optimize PFA outcomes.

## Funding

The authors have nothing to report.

## Ethics Statement

This study was conducted in accordance with the Declaration of Helsinki, the Good Clinical Practice, the corresponding national laws, the contemporaneous guidelines, and according to the current state of science and technology. Approval was granted by the Institutional Review Board.

## Consent

All the participants provided informed consent.

## Conflicts of Interest

The authors declare no conflicts of interest.

## Data Availability

The data that support the findings of this study are available from the corresponding author upon reasonable request.
